# Thiolation of Myco-Synthesized Fe_3_O_4_-NPs: A Novel Promising Tool for *Penicillium expansium* Laccase Immobilization to Decolorize Textile Dyes and as an Application for Anticancer Agent

**DOI:** 10.3390/jof8010071

**Published:** 2022-01-11

**Authors:** Hamed M. El-Shora, Aiah M. Khateb, Doaa B. Darwish, Reyad M. El-Sharkawy

**Affiliations:** 1 Department of Botany, Faculty of Science, Mansoura University, Mansoura 35511, Egypt; d_darwish@mans.edu.eg; 2 Department of Medical Laboratory Technology, College of Applied Medical Sciences, Taibah University, Medina 41411, Saudi Arabia; akhateb@taibahu.edu.sa; 3 Botany and Microbiology Department, Faculty of Science, Benha University, Benha 13511, Egypt; r.m.elsharkawy@fsc.bu.edu.eg

**Keywords:** *Penicillium expansium*, thiolated nanosupport, immobilized laccase, biocatalyst, decolorization, cytotoxicity

## Abstract

Environmental pollution due to the continuous uncontrolled discharge of toxic dyes into the water bodies provides insight into the need to eliminate pollutants prior to discharge is significantly needed. Recently, the combination of conventional chemotherapeutic agents and nanoparticles has attracted considerable attention. Herein, the magnetic nanoparticles (Fe_3_O_4_-NPs) were synthesized using metabolites of *Aspergillus niger*. Further, the surfaces of Fe_3_O_4_-NPs were functionalized using 3-mercaptoproionic acid as confirmed by XRD, TEM, and SEM analyses. A purified *P. expansum* laccase was immobilized onto Fe_3_O_4_/3-MPA-SH and then the developed immobilized laccase (Fe_3_O_4_/3-MPA-S-S-laccase) was applied to achieve redox-mediated degradation of different dyes. The Fe_3_O_4_/3-MPA-S-S-laccase exhibited notably improved stability toward pH, temperature, organic solvents, and storage periods. The Fe_3_O_4_/3-MPA-S-S-laccase exhibited appropriate operational stability while retaining 84.34% of its initial activity after 10 cycles. The catalytic affinity (K_cat_/K_m_) of the immobilized biocatalyst was increased above 10-fold. The experimental data showed remarkable improvement in the dyes’ decolorization using the immobilized biocatalyst in the presence of a redox mediator in seven successive cycles. Thus, the prepared novel nanocomposite-laccase can be applied as an alternative promising strategy for bioremediation of textile wastewater. The cytotoxic level of carboplatin and Fe_3_O_4_-NPs singly or in combination on various cell lines was concentration-dependent.

## 1. Introduction

Environmental pollution has been considered a daunting challenge in the twenty-first century, mainly in underdeveloped and developing countries [1,2,3]. The rapid industrialization and technological development have led to the rapid discharge of untreated toxic textile dyes and harmful contaminants into the water bodies [4,5]. The cosmetic, food, pharmaceutical, textile, printing, and leather industries are discharging recalcitrant pollutants into natural water bodies. However, water pollution produced through the discharge of untreated toxic/recalcitrant textile dyes into water bodies has severe harmful effects on the continuation of life in the specific biosphere [6,7].

Coagulation, oxidation, electrochemical precipitation, flocculation, nano-filtration, and adsorption are among the considered numerous physical and chemical technologies which have been applied either singly or in combination for the eradication of recalcitrant textile dyes [5,8,9].

The above methods mentioned for wastewater clean-up are quite expensive, limiting their actual application at large industrial scales [6,10]. In recent decades, nanocomposite, nanoparticles, and biosorbents have been widely employed for textile dyes adsorption. However, researchers are nowadays focused on the performance of advanced adsorbents for treating waste streams. 

The enzymes immobilized on a magnetic nanosupport offer different advantages, such as low operation price, simple separation using an external magnet, reduction in enzyme diffusion, higher stability, higher adsorption sites, and consequently, a higher quantity of binding enzymes. 

Furthermore, the presence of different functional groups on the nanoparticle surface has an extra vital advantage which facilitates the functionalization (surface modification) of the nanosupport using various synthetic and natural compounds, such as chitosan and 3-mercaptoproionic acid [11,12].

Laccase (E.C. 1.10.3.2) is one of the highest oxidoreductases enzymes with regard to its application in the remediation of environmental pollutants. Laccases catalyze the oxidation of the target pollutant coupled to the molecular oxygen reduction to water [5,13,14]. The oxidation rate of pollutants by laccase is improved through the performance of small molecular weight redox mediators [6].

One such novel strategy is the immobilization of laccase on a thiolated magnetic nanosupport which has been carried out using 3-mercaptoproionic acid (3-MPA) covalent-binding. The -SH of the 3-MPA loaded onto Fe_3_O_4_ (Fe_3_O_4_/3MPA-SH) is covalently bound to the -SH of the laccase, producing an appropriate stable disulfide (-S-S-) bond at pH 5.0 [1,9,15]. Hence, this study chiefly attempts the laccase immobilized on the thiolated magnetic nanocomposite which can substantially offer applicable nanosupports for biocatalyst immobilization performances. 

Carboplatin is commonly employed in chemotherapy for different cancer cells [16,17]. A combination of nanoparticles, especially Fe_3_O_4_-NPs of a super-magnetic nature, with commonly established chemotherapeutic agents is considered a new platform for performing new therapeutic approaches [18]. 

The novelty of the present study is the laccase immobilization on the stable, reusable, and sustainable thiolated nanosupport using a new immobilization system [11,12]. In the present study, the Fe_3_O_4_-NPs have been synthesized using biomolecules of *A. niger* as reducing and coating agents. The thiolated ligand (3-MPA) was employed for the functionalization of the nanoparticles. The characterization of the morphology and structure of the nanosupport was carried out by FTIR, XRD, SEM, and TEM analyses. *Penicillium expansum* is the most common and economically important postharvest fruit rot pathogen that causes blue mold. *P. expansium* EG-MR15 showed the highest laccase activity among the investigated fungal isolates. Such strain was used as a producer for the enzyme in our work. The purified *P. expansium* laccase was covalently immobilized onto Fe_3_O_4_/3-MPA-SH. Finally, the newly developed immobilization system (Fe_3_O_4_/3-MPA-S-S-Lac) was checked for its possible catalytic degradation of different toxic textile stains, i.e., Methyl Orange, Brilliant blue, Remazol Brilliant Blue R, and Reactive Black-5. The cytotoxic potentiality of carboplatin and nanoparticle was investigated either alone or in combination on different cancer cell lines.

## 2. Materials and Methods

### 2.1. Materials and Chemicals

3-ethyl benzothiazoline-6-sulfonic acid (ABTS) and 3-mercaptopropanoic acid (HSCH_2_CH_2_CO_2_H) were brought from Sigma–Aldrich (St. Louis, MO, USA). Other chemicals of analytical grade, FeCl_3_·6H_2_O, FeSO_4_·7H_2_O and 1-hydroxybenzotriazole (HBT) were obtained from Sigma Chemical Co., Cairo, Egypt. Methyl Orange (MO), Reactive Black-5 (RB-5), Brilliant blue (Bb), and Remazol Brilliant Blue R (RBBR) were purchased from Sigma–Aldrich. All other reagents and chemicals used in the present research were of analytical grade. MCF-7 (human breast cancer cells), HepG2 (human hepatocellular carcinoma cells), and A549 cell lines were brought from ATCC via the holding company for biological products and vaccines (VACSERA), Cairo, Egypt.

### 2.2. Bio-Synthesis of Thiol-Functionalized Magnetic Nanoparticles

Magnetite (Fe_3_O_4_-NPs) nanoparticles were prepared according to [11] with definite modifications. Briefly, the *A. niger* (MW390925.1) used in this work was freshly inoculated into the Czapek’s-Dox broth [19]. After incubation at 28 °C for 5 days, the developed pellets were excluded by filtration. Subsequently, the filtrate was then centrifuged at 10,000× *g* for 20 min and 4 °C. The clear supernatant was used for the biosynthesis of Fe_3_O_4_-NPs. The metallic precursor solution was attained by mixing FeSO_4_·7H_2_O and FeCl_3_·6H_2_O in a 1:1 ratio. 

For Fe_3_O_4_-NPs biosynthesis, an aqueous metal solution (50 mL) was mixed with the same volume of fungal supernatant as the reducing and coating agent. The contents were heated at 60 °C and stirred magnetically for 2 h. The mixture pH was retained at 12 until the formation of a black color. The Fe_3_O_4_-NPs were then harvested by centrifugation for 30 min at 5000 rpm and 4 °C, washed thrice with distilled water as well as ethanol. The magnetic nanoparticles were dehydrated at 60 °C for 12 h. For functionalization, a desirable amount of magnetic nanoparticles (5 g) was mixed with 2.3 g of 3-mercaptopropanoic acid (3-MPA) by ultra-sonication in 50 mL distilled water for 12 h at ambient temperature. The preparation was kept at pH 8.0 using NaOH (0.1 M). The obtained black particles were then collected and washed with ethanol to become neutral pH. Furthermore, the black particles were pooled and dried in an oven for 20 min at 80 °C and then used for laccase immobilization [1,11,20].

### 2.3. Characterization of Functionalized Magnetic Nanocomposite

The prepared functionalized magnetic nanoparticles (Fe_3_O_4_/3-MPA-SH) were characterized by various instruments, including X-ray powder diffraction pattern (MiniFlex 300/600 X-ray, USA), Fourier transform infrared (FT-IR) spectroscopy, Scanning electron microscope (SEM, JEOL JSM-6510LV microscope, JEOL Ltd., Tokyo, Japan), Transmission electron microscope (TEM, JEOL JEM-1010 microscope), and Energy dispersive X-ray spectrometer (EDX).

### 2.4. Biotechnological Applications of Fe_3_O_4_/3-MPA-SH in Laccase Immobilization 

#### 2.4.1. Screening for the Most Potential Laccase Producer Fungal Isolate 

The fungal isolates used in this work were freshly isolated from different soil samples (Benha, Egypt), and their ability to produce laccase was detected using a modified medium (MM) containing 2.0 g/L corn steep liquor; 0.07 g/L KCl; 1.2 g/L NH_4_H_2_PO_4_; 0.5 g/L MgSO_4_·7H_2_O; 0.1 g/L FeSO_4_·7H_2_O supplemented with 0.5 mM (ABTS) on a rotary shaker (120 rpm) for 5 days at 28 °C. The mycelial pellets were excluded, washed, and homogenized in phosphate buffer (pH 5.0, 50 mM) for 30 min. Subsequently, the homogenate was centrifuged for 20 min at 10,000× *g* and 4 °C. The clear supernatant represented crude extract of laccase and the enzyme activity was measured according to [6].

#### 2.4.2. Laccase Activity Assay and Protein Determination

The activity was assessed spectrophotometrically at 420 nm using 0.5 mM ABTS with the extinction coefficient (ε = 36,000 M^−1^ cm^−1^). The unit of laccase activity was expressed as the amount of enzyme needed for oxidizing 1 µM ABTS per min under standard assay conditions. The protein content was estimated according to the Bradford assay method [21], and compared to bovine serum albumin. 

#### 2.4.3. Identification and Deposition of the Most Potential Laccase-Producing Isolate

The potent isolate producing laccase used in this work was identified according to the observation of its morphological and molecular characteristics as *Penicillium expansium* [22]. The sequence ITS region was analyzed and then aligned with its closely related sequences at the NCBI server (http://www.ncbi.nlm.nih.gov/BLAST, accessed on 5 December 2021). The FASTA sequences of the GenBank similar sequences were imported into the MEGA 10.0 portal. Then, the sequences were then aligned for the multiple sequence alignment with the ClustalW muscle algorithm. The phylogenetic tree was generated using the Neighbor-Joining method with a confidence level of 1000 bootstrap replication [6,14].

#### 2.4.4. Extraction, Purification, and Molecular Homogeneity of Laccase

Extraction of laccase was performed according to [6]. Briefly, one ml (10^6^ spore/mL) of *P. expansum* EG-MR15 (Accession number OL719228.1) was inoculated into 50 mL of MM medium supplemented with 0.5 mM ABTS, incubated at 28 °C under shaking (120 rpm) for 5 days. After incubation, the fungal pellets were excluded by filtration. The obtained filtrate was centrifuged at 10,000× *g* for 20 min under 4 °C. The supernatant was employed as the crude enzyme and kept at −20 °C for further work.

The laccase preparation was purified by salting out, DEAE-cellulose column (ion-exchange chromatography), and Sephadex G-200 column (gel-filtration chromatography), and later used for the assay. Briefly, the crude enzyme extract was supplemented by ammonium sulfate (75% saturation) with soft stirring at 4 °C for 60 min, centrifuged at 10,000 rpm for 20 min. The precipitate was dialyzed at 4 °C for 24 h versus 20 mM phosphate buffer (pH 7.0) prepared with NaCl (150 mM). The developed concentrated laccase was applied onto a DEAE-cellulose column, after pre-equilibration with the same buffer. The active fractions were collected, dialyzed, and downstream purified using a Sephadex G-200 column. The activity of laccase was assayed according to [6]. The SDS-PAGE was used to characterize the molecular homogeneity of the purified enzyme [23]. The markers consisted of carbonic anhydrase (29 KDa), aldolase (44 KDa), ovalbumin (60 KDa), BSA (84 KDa), and acid phosphatase (100 KDa).

#### 2.4.5. Immobilization of Laccase on a Thiolated Functionalized Magnetic Nanosupport

The activated nanoparticles (10 mg) were sonicated for 15 min in 20 mL of Na acetate buffer (pH 5.0, 0.1 M) before adding laccase (Lac). Subsequently, the purified laccase (1 mg/mL, 100 U/mg protein) in the same acetate buffer (10 mL, pH 5.0, 0.1 M) was added and incubated, and slowly stirred at ambient temperature for 24 h. The conjugated laccase onto thiol-activated nanosupport (Fe_3_O_4_/3-MPA-S-S-laccase) was isolated using an external magnet and washed many times using Na acetate buffer (0.1 M) until the enzyme activity disappeared from the washing solution. The laccase loading ability of the nanosupport was evaluated using different initial Lac concentrations (0.25–1.25 mg/mL). The Fe_3_O_4_/3-MPA-S-S-laccase was subjected to activity recovery percentage and biocatalyst loading capacity determination. Fe_3_O_4_/3-MPA-S-S-laccase was preserved in the same buffer at 4 °C for further use. Control beads were prepared without adding the enzymatic preparation into Fe_3_O_4_/3-MPA-SH [1].

The laccase activity was detected according to [6] with some modifications. Briefly, the reaction mixture contained 0.9 mL of Na-acetate buffer (0.1 M, pH 5.0), 1 mL of 0.5 mM ABTS, and 0.1 mL of soluble laccase (1 mg/mL), or 0.1 g of Fe_3_O_4_/3-MPA-S-S-laccase. The reaction mixtures were kept for 20 min at 28 °C. The activity recovery was calculated using the following equation [1].

$$\mathrm{Activity}\;\mathrm{recovery}\;(\%)=\frac{A_{I}}{A_{Fr}}\times 100$$

where $A_{I\;}$ is the immobilized laccase activity, $A_{Fr\;}$ is the soluble laccase activity before immobilization. The level of protein bound to nanocomposite was detected by subtracting the recovered protein in the washing buffer from the protein applied for immobilization [24,25]. Laccase loading capacity was assessed by the following equation [1].

$$\mathrm{Biocatalyst}\;\mathrm{loading}\;\mathrm{capacity}\;(\mathrm{mg}/g)=\frac{\left(C_{i}-C_{f}\right)\;V}{M}\times 100$$

where $C_{i\;}$ is the initial protein concentration used for immobilization (mg/L), $C_{f\;}$ is the final protein concentration post immobilization (mg/L), *V* is the solution volume (L), and *M* is the nanocomposite weight (g). Furthermore, the properties of Fe_3_O_4_/3-MPA-S-S-laccase were evaluated. 

#### 2.4.6. Characterization of Free Laccase and Fe_3_O_4_/3-MPA-S-S-Lac

##### pH Optima and pH Stability

The optimum pH of the free laccase and the enzyme anchored on nanocomposite was determined under standard assay conditions and using ABTS as a substrate in various pH buffers (0.1 M of sodium acetate buffer, 2.0–5.0; phosphate buffer, 6.0–7.0; Tris-HCl buffer 8.0–9.0). The highest value of laccase activity was defined as 100%. The pH stability was determined by preincubating free laccase and Fe_3_O_4_/3-MPA-S-S-laccase in respective buffers at ambient temperature for 60 min. The residual activity was assayed using ABTS in standard conditions. 

##### Optimum Temperature and Thermal Stability

The optimum temperatures of free laccase and Fe_3_O_4_/3-MPA-S-S-laccase were investigated by incubating the reaction mixture at different temperatures (30–70 °C) using 0.5 mM ABTS in optimal pH. For the thermostability assay, the free and immobilized preparations were separately pre-incubated at the selected temperatures for 180 min. Laccase activity was determined in standard conditions at 40 °C with ABTS. 

##### Determination of K_m_ and V_max_

The maximum velocity (V_max_) and Michaelis constant (K_m_) values of the free and immobilized laccase were estimated by the Lineweaver-Bürk plot [26] using different concentrations (0.2–0.8 mM) of ABTS (non-phenolic substrate) and catechol (phenolic substrate) in sodium acetate buffer (pH 5.0, 0.1 M) at 40 °C.

##### Effect of Different Organic Solvents on Enzyme Stability

The free laccase and Fe_3_O_4_/3-MPA-S-S-laccase were incubated with various organic solvent concentrations (10–50% *v/v*) for 24 h at room temperature. The activity of the enzyme was subsequently determined in standard assay conditions. In parallel, the enzymatic preparations without any organic solvent were performed under the same conditions to represent the controls. The residual laccase activity was determined, relative to the corresponding control, which was defined as 100%. 

##### Operational Stability (Reusability)

The reusability of Fe_3_O_4_-NP_s_/3-MPA-S-S-laccase was investigated for ten consecutive cycles using 0.5 mM ABTS. Briefly, 5 mL of fresh substrate solution was mixed with 10 mg of the Fe_3_O_4_/3-MPA-S-S-laccase in 5 mL of Na acetate buffer (pH 5.0, 0.1 M) for 30 min with persistence agitation. After each cycle, the immobilized laccase was collected using a magnet and rinsed twice by Na-acetate buffer to remove the remaining ABTS and then followed by repeated trials with a fresh aliquot of the substrate. The initial activity of the Fe_3_O_4_/3-MPA-S-S-laccase was defined as 100%.

##### Storage Stability

The free laccase and Fe_3_O_4_/3-MPA-S-S-laccase were stored in Na acetate buffer (0.1 M, pH 5.0) at 4 °C and room temperature for 40 days. The residual activity was determined at intervals of 1, 5, 10, 15, 20, 25, 30, and 40 days in standard conditions. The free and immobilized laccase activities were assayed by ABTS as substrate. The residual activity of the fresh enzyme was assigned to be 100%.

##### Decolorization of Synthetic Dyes

The decolorization efficiency of target pollutants (synthetic dyes) from aqueous solution by free laccase, Fe_3_O_4_/3-MPA-SH, and Fe_3_O_4_/3-MPA-S-S-laccase was investigated. The synthetic dyes, namely methyl orange (MO), Reactive Black 5, Remazol Brilliant Blue B (RBBR), and (RB-5) Brilliant blue (Bb) were chosen as model dyes for testing the decolorization capacity in the present batch experiments. Stock solutions (0.2% *w/v* in water) of the tested dyes were kept at ambient temperature in the dark. Assays of synthetic dyes’ decolorization was conducted using 30 units of 15 mg of Fe_3_O_4_/3-MPA-S-S-laccase in Na acetate buffer (0.1 M, pH 5.0) and 1 mM redox mediator (1-hydroxybenzotriazole).

At the maximum visible λ of each dye, the concentrations of the tested dyes were chosen in order to attain absorbance around 1.0 units. In parallel, controls were performed with 15 mg of Fe_3_O_4_-NP_s_/3MPA-SH (without enzyme) and another with the same volume (units) of the free enzyme as the immobilized counterpart. The reaction was performed in the dark at pH 5.0, temperature 40 °C with continuous agitation to attain proper oxygenation for 6, 12, 24, 48, and 96 h. The corresponding concentration of dye before and after batch trials was monitored by a UV/visible spectrophotometer. The decolorization efficiency was defined in terms of percentage [6]. The magnetic nanocomposites were gathered by a permanent magnet after each experiment. 

##### Reusability Assessment of Fe_3_O_4_/3-MPA-S-S-Laccase

The reusability potential of Fe_3_O_4_/3-MPA-S-S-laccase for decolorization of four toxic textile dyes was assessed for several cycles each of 24 h. After the end of each cycle, the Fe_3_O_4_-NP_s_/3MPA-S-S-Lac was collected by a magnet and subsequently washed with Na acetate buffer (pH 5.0). The decolorized solution was then substituted with a new dye solution to carry out the further cycles. The immobilized enzyme activity in the first cycle was assigned as 100% and the relative activity was computed for the repetitive degradation cycles. 

### 2.5. Cytotoxic Effect

The cytotoxic potentiality of carboplatin and Fe_3_O_4_-NPs alone or in combination (carboplatin and Fe_3_O_4_-NPs) on the HepG2 (human hepatocellular carcinoma cells), MCF-7 (human breast cancer cells), and A549 cell lines was evaluated according to MTT (3-[4,5-dimethylthiazol-2-yl]-2,5-diphenyltetrazolium bromide) reduction assay analysis [27,28].

In brief, the cells were plated before the addition of the tested compound in a sterile 96-microtiter plate and incubated for 24 h at 37 °C. The tested substances were supplemented into a growth medium containing 1 × 10^4^ cells/well to attain different concentrations of carboplatin (5, 10, 15, 20 µM), Fe_3_O_4_-NPs (20, 40, 60, 80 µg/mL), and the combination of carboplatin and Fe_3_O_4_-NPs.

The test was performed in a total volume of 100 µL and the treated cells were sustained in an incubator for 24 h at 37 °C. The MTT solution (10 µL, 5 mg/mL) was introduced per well and incubated for 3 h under 5% CO_2_ at 37 °C. The media were discarded and the formed purple formazan crystals were suspended using DMS (100 µL). Cells without any treatment were considered as positive control, while the medium only was negative control. Optical density after 15 min was determined at 570 nm by a microplate reader (680 XR reader, Bio-Rad).

### 2.6. Statistical Analysis 

All runs were repeated three times, and the obtained experimental data were represented as the mean value of each trial ± standard deviation (SD). Cytotoxic assay results were examined for the normality test and then one-way ANOVA was performed at a significant level of *p* < 0.05, then a Tukey’s post-hoc test was carried out. Statistical analyses were accomplished by the Statistical Package for Social Sciences (SPSS) version 25 (IBM, Armonl, NY, USA).

## 3. Results and Discussion

### 3.1. Synthesis

**Figure 1:**
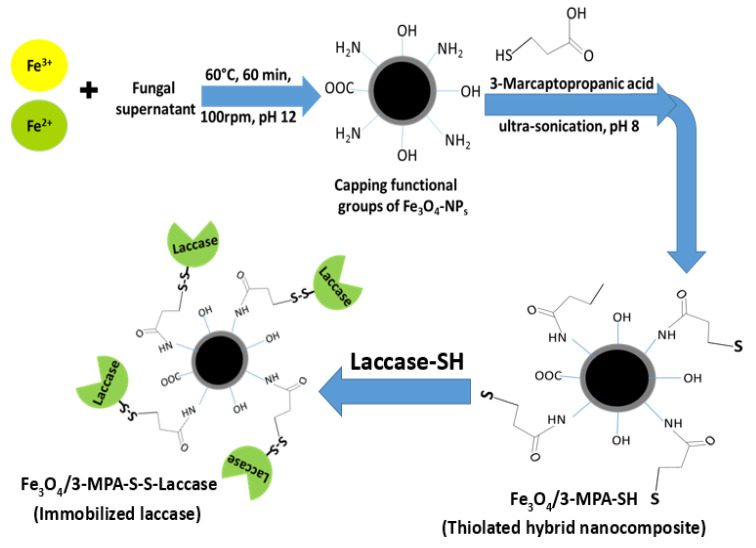
Schematic diagram showing the biosynthesis of magnetic nanocomposite using metabolites of *A. niger*, thiolation of nanocomposite using 3-mercaptopropanoic acid, and the immobilization process of *P. expansum* laccase on a thiolated hybrid nanocomposite.

The overall synthesis process of Fe_3_O_4_-NPs, Fe_3_O_4_/3-MPA-SH and Fe_3_O_4_/3-MPA-S-S-laccase is illustrated in Figure 1. The metallic precursors (Fe^2+^:Fe^3+^) were firstly mixed with *A. niger*-fungal filtrate in order to synthesis magnetic nanocomposite which was capped with different functional groups. Further, the developed Fe_3_O_4_-NPs were surface modified with 3-MPA. It is well recognized that Fe_3_O_4_/3-MPA shows ubiquitous –SH groups on its surface. The thiolated nanocomposite (Fe_3_O_4_/3-MPA-SH) was covalently bound to the –SH groups of laccase, fabricating Fe_3_O_4_/3-MPA-S-S-Lac (immobilized laccase) through the formation of a disulfide bond. Similar results for the production of immobilized laccase through covalent bonding to thiolated supports have been described earlier [1,9]. 

### 3.2. Characterization of the Nanosupport for Laccase Immobilization 

**Figure 2:**
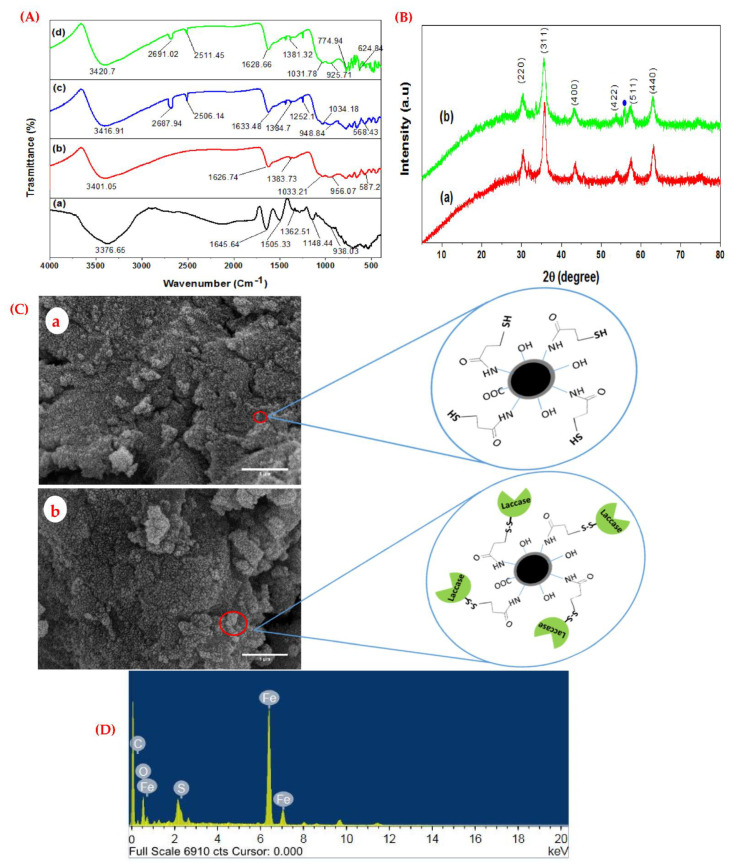
(**A**) The FTIR spectrum of (**a**) fungal extract; (**b**) biosynthetic Fe_3_O_4_ nanoparticles; (**c**) 3-MPA functionalized Fe_3_O_4_-NPs; (**d**) Fe_3_O_4_/3-MPA-S-S-Lac, (**B**) X-ray diffraction pattern of (**a**) the biosynthetic Fe_3_O_4_ nanoparticles; (**b**) Fe_3_O_4_/3-MPA-SH. In the spectrum, (**b**) corresponds to the SH-functionalized magnetite nanoparticles. Herein, the filled circle indicates the covering of the thiol group onto Fe_3_O_4_-NP, (**C**) SEM images of the Fe_3_O_4_ nanoparticles (**a**) and Fe_3_O_4_/3-MPA-SH (**b**) (inset the proposed schematic structure of the immobilized laccase on a thiolated hybrid nanocomposite), (**D**) EDX of Fe_3_O_4_/3-MPA-SH.

Fungal biosynthesis of Fe_3_O_4_-NPs and 3-MPA capping of Fe_3_O_4_-NPs was evidently verified from FTIR (Figure 2A). Various peaks were observed in the spectral range from 400 to 4000 cm^−1^, corresponding to the plausible existence of different functional groups on the surface of the biosynthetic magnetic nanoparticles. A characteristic broad band was detected from 3420 to 3000 cm^−1^ which might correspond to the overlapping O–H, N–H, and aromatic hydrogen stretching vibration (Figure 2A(a). The development of inter- and intra-molecular hydrogen bonds is the possible reason for the peak shift and considerable peak width (Figure 2A(b,c)), as reported by [11]. A well-characterized peak at 1645 cm^−1^ was assigned to the C=N or C=O stretching vibration of amide or acid derivatives, which was shifted to a lower wavelength (Figure 2A(b,c)). A peak at 1148 cm^−1^ was assigned to the C–O stretching vibration. A very low-intensity peak at 1033 cm^−1^ could be associated with the Fe–OH vibration. The absorption peak observed at 587 cm^−1^ was attributed to the Fe–O–Fe stretching vibration (Figure 2A(b)). Five characteristics peaks were observed at 3416 cm^−1^ (overlapped N–H and O–H stretching vibration), 2687, and 2506 cm^−1^ (S–H stretching vibration), 1633 cm^−1^ (COOH), and 568 cm^−1^ (CSH stretching vibration), indicating the successful surface modification of the magnetic nanoparticles using 3-MPA. The presence of the carboxylic group and thiol group on the surface of the magnetic nanoparticles, confirmed the participation of fungal metabolites in the reducing and capping processes of Fe_3_O_4_-NPs and the smooth capping of 3-mercaptopropionic acid onto magnetic nanoparticles as reported by [11,29].

Upon immobilization of laccase onto Fe_3_O_4_/3-MPA-SH, two new peaks were, respectively, observed at 774 cm^−1^ and 624 cm^−1^, hinting the S–S and C–S when compared with Fe_3_O_4_-NPs and Fe_3_O_4_/3-MPA-SH (Figure 2A(d)). During the immobilization process, it is clear that the thiolated magnetic nanoparticles reacted with the thiol group on the laccase side, forming a strong disulfide bond (–S–S–). The performance of a strong disulfide bond displayed an excellent method for immobilization. Concurring with certain research reports, the laccase was conjugated onto the thiolated chitosan composite [1,9,30]. Overall, the FTIR spectra proved the participation of the fungal extract containing biomolecules on the surface of the magnetic nanoparticles, the functionalization by 3-MPA, and are consistent with [11,20,31].

The crystalline pattern of Fe_3_O_4_-NPs and 3-MPA/Fe_3_O_4_-NP_s_ was evaluated through the XRD analysis. Similar diffraction peaks were obtained before and after thiolation, as illustrated in Figure 2B, hinting at the crystalline nature after the smooth capping of 3-mercaptopropionic acid onto Fe_3_O_4_-NPs. Six characteristic diffraction peaks (Figure 2B(a)) were observed at 2θ = 30.4°, 35.7°, 43.5°, 54.07°, 57.3°, and 63.08°, respectively, which can be indexed to the plans of pure Fe_3_O_4_-NPs at (220), (311), (400), (422), (511), and (440). The peaks at 2θ = 35.7° and 63.08° were essentially showing the existence of iron oxide. The obtained magnetic nanoparticles showed an average crystallite size of ~7.8 nm. A new characteristic peak was observed at 2θ = 56.1°, illustrating the successful capping/coating of 3-MPA on magnetic nanoparticles (Figure 2B(b)). The brooding and weak intensity principally indicates the nano-size of the magnetic particles. These findings are in agreement with those described by [12,32].

The morphological observations of the Fe_3_O_4_-NPs and Fe_3_O_4_/3-MPA-SH nanosupport were determined by SEM as shown in Figure 2C. The magnetite nanoparticles exhibited a uniform spherical structure with homogenous distribution (Figure 2C(a)). The hybrid nanocomposite (Fe_3_O_4_/3-MPA-SH) was mostly agglomerated as a result of the incorporation of 3-MPA ligands. The elemental analysis using EDX showed the intense peaks of iron, oxygen, and sulfur (Figure 2D). Almost similar results with respect to the surface morphology and elemental distribution of the thiolated magnetite nanocomposite were detected by [1,11].

**Figure 3:**
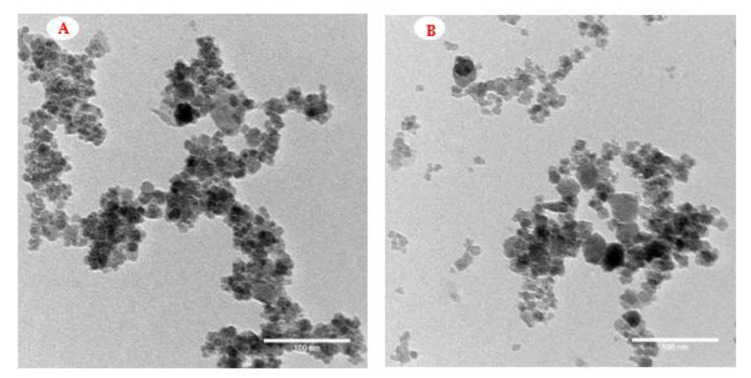
TEM analysis of Fe_3_O_4_-NPs (**A**) and Fe_3_O_4_/3-MPA-SH (**B**). Scale bar = 100 nm.

The TEM analysis of the Fe_3_O_4_-NPs and Fe_3_O_4_/3-MPA-SH nanocomposite is shown in Figure 3. The obvious spherical and quasi-polyhedral structure of Fe_3_O_4_-NPs can be observed. The 10–18 nm size of the particles was in remarkable agreement with the results detected from the XRD analysis. The granular size was increased after the performance of 3-MPA without significant fluctuations in the granular morphology (Figure 3B). The size of Fe_3_O_4_/3-MPA-SH nanocomposite was found to be 16–20 nm. Overall, the FT-IR, SEM, EDX, and TEM analyses clearly illustrated the successful biosynthesis of Fe_3_O_4_-NPs using the fungal extract and the capping of Fe_3_O_4_ by 3-MPA.

### 3.3. Screening for the Most Potent Laccase Producing Isolate

Twenty-five fungal isolates were grown on modified medium supplemented with 0.5 mM ABTS for testing their laccase productivity. The developed fungal isolates showed a plausible fluctuation in the Lac activity, of which six isolates displayed as highly active. The highest Lac activity was determined for *P. expansium* EG-MR15, compared to the other fungal isolates.

**Figure 4:**
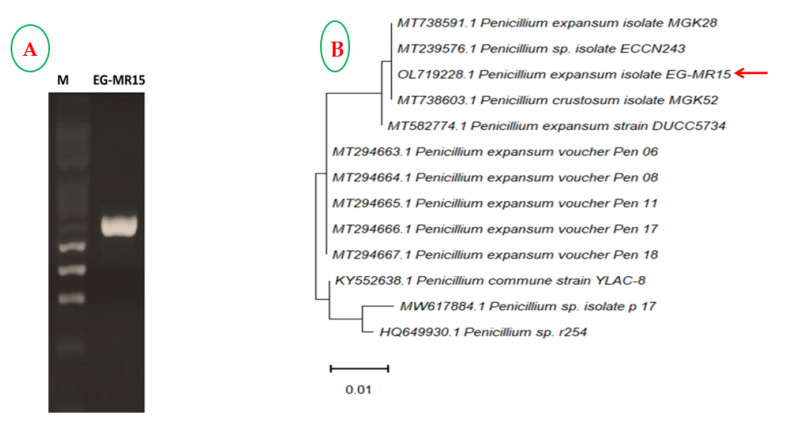
(**A**) Molecular identification using the PCR amplicon of region 18S-ITS1-5.8S-ITS2-28S; Lane M: 1 kb molecular marker and Lane of EG-MR15 (~500 bp). (**B**) Molecular phylogenetic tree of *P. expansium* EG-MR15 with closely related BLAST resulted in sequences constructed by the Neighbor-Joining method. The bar length denotes 0.01 substitutions for each nucleotide site. The isolate in the present study is indicated by the red arrow.

The morphological characterization of the most potent fungal isolate producing laccase was approved based on the sequence of the ITS region (Figure 4). The purified amplicon was sequenced and then undergo a non-redundantly BLAST search in the NCBI database. The sequence was deposited to the NCBI database under accession number OL719228.1. The phylogenetic tree of the rDNA sequence was generated (Figure 4B) using the Neighbor-Joining method with bootstrap replication of 1000. The isolate *P. expansium* EG-MR15 had a 100% similarity with the isolates of *P. expansium* with accession number MT738591.1, MT239576.1, and MT738603.1, with zero E-value and 99% query coverage. 

### 3.4. Production of Purified Laccase from P. expansium

The productivity of laccase by the culture of *P. expansium* EG-MR15 was assessed by growing the fungal strain on a medium supplemented with 0.5 mM ABTS. Laccase was extracted and purified using ammonium sulfate (75%), DEAE-cellulose column, and Sephadex G-200 column. For each purification step, the most active fractions were pooled, concentrated, employed in the subsequent purification step, and later used for assay as mentioned previously. The specific activity of laccase by ammonium sulfate was augmented by nearly 3.18-fold associated with the crude enzyme with an overall yield of 69%. Using the DEAE-cellulose column, the specific activity of Lac was increased by nearly 14.76-fold related to the crude enzyme with a 42% overall yield. The specific activity of laccase by gel-filtration chromatography was practically improved by about 33.05-fold with an overall yield of 27%, compared with the crude enzyme. The overall purification profile of laccase from *P. expansium* is briefly summarized in Table 1. Hence, the purified laccase was employed for the preparation of enzyme-immobilized magnetic beads. 

**Figure 5:**
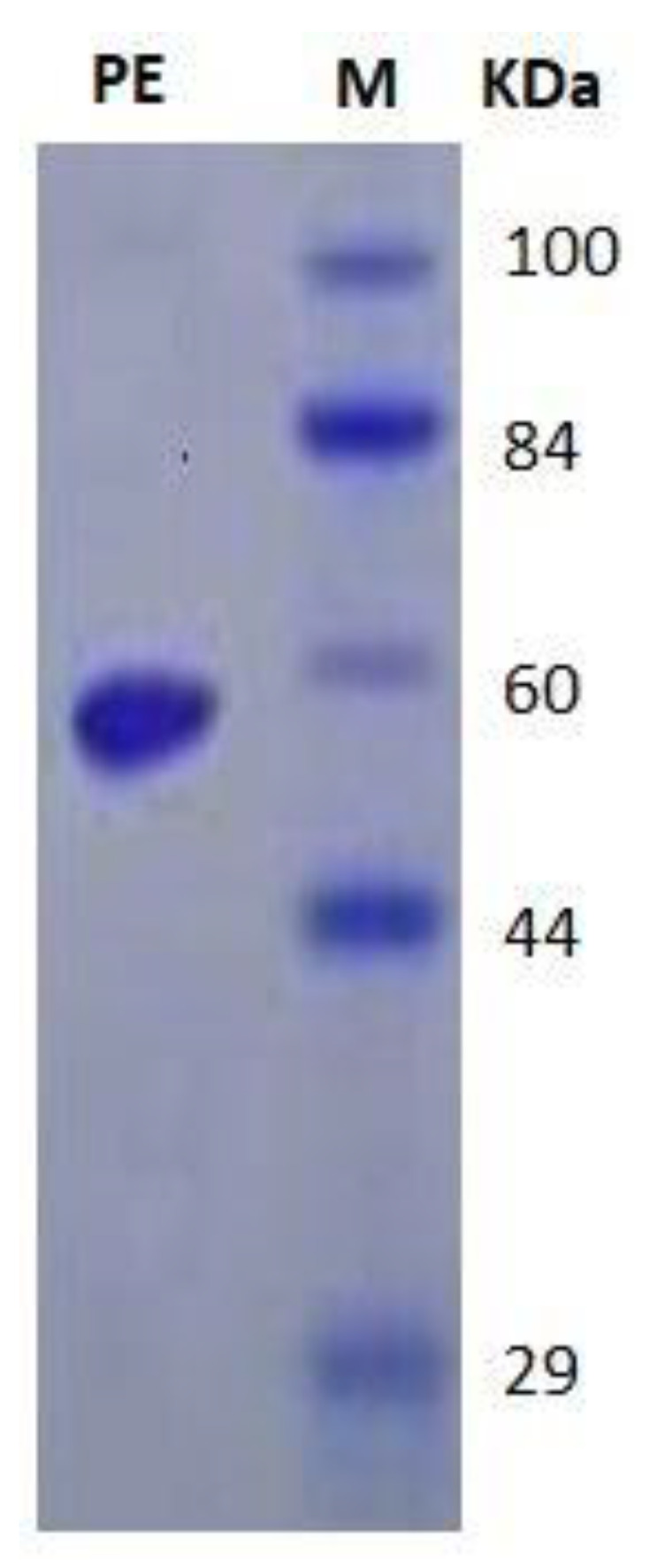
SDS-PAGE of the purified laccase from *P. expansium* EG-MR15. Lane M: Marker protein and Lane PE: Purified laccase enzyme.

The molecular homogeneity of purified *P. expansium* laccase was investigated by SDS-PAGE [14]. A single protein band of molecular mass ~60 KDa was determined (Figure 5). A previous study described Lac purification from *Coriolopsis gallica* with a 4.9-fold increase and an overall yield of 60.6% [33]. The molecular mass of laccase was coincident with most laccases that are monomeric proteins, as confirmed with non-denaturing PAGE, with a molecular weight of ~50 to 80 KDa [6,34,35,36]. 

### 3.5. Immobilization of Laccase on Fe_3_O_4_/3-MPA-SH

In order to authenticate the binding capacity of laccase to the thiolated magnetic nanosupport, a comparative study between free laccase and immobilized laccase (Fe_3_O_4_/3MPA-S-S-Lac) was conducted. The reaction was performed using 0.1 mL of free preparation in Na acetate buffer (pH 5.0, 0.1 M) or 10 mg Fe_3_O_4_-3MPA-S-S-Lac in Na acetate buffer (pH 5.0, 0.1 M). The activity of soluble laccase from *P. expansum* toward ABTS was 25 U/mg protein. The results showed that the laccase immobilized on the thiolated-nanosupport, and the optimum concentration was 10 mg, which was the optimum with 92% activity recovery. Furthermore, the loading of laccase onto the surface of Fe_3_O_4_/3MPA-SH was assessed using various initial laccase concentrations (0.25–1.25 mg/mL).

**Figure 6:**
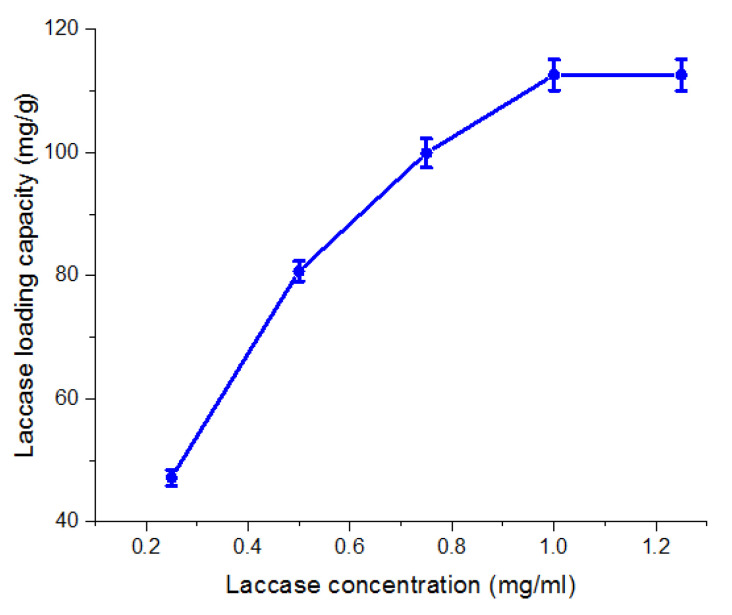
Effect of different laccase initial concentrations on the immobilization ability over thiolated magnetic nanoparticles.

The results in Figure 6 clearly illustrate the optimum loading capacity of laccase, which was determined at 1 mg/mL initial laccase concentration, hinting at the best occupation of the Fe_3_O_4_/3-MPA-SH immobilization sites. The laccase loading capacity (mg/g) using the above-mentioned nanosupport was superior when compared with other latest nanosupports used for laccase immobilization using different cultures (Table 2). The thiolation (-SH) of Fe_3_O_4_-NPs delivered unique binding sites for the immobilization of laccase. The -SH group of laccase is covalently bound to the thiol group located over the Fe_3_O_4_/3-MPA to produce the disulfide bond as speculated, which in turn remarkably improved the biocatalyst loading efficiency over the thiolated magnetic nanoparticles. Several researchers described a laccase immobilization over thiolated supports [1,9,15]. 

### 3.6. Biochemical Characterization of the Free and Immobilized Laccase 

After the confirmation of the successful covalent binding of laccase over the thiolated modified magnetic nanocomposite and the biocatalyst loading capacity determination, the developed Fe_3_O_4_/3-MPA-S-S-Lac was employed for extended characterization studies. 

#### 3.6.1. pH Optima and pH Stability

**Figure 7:**
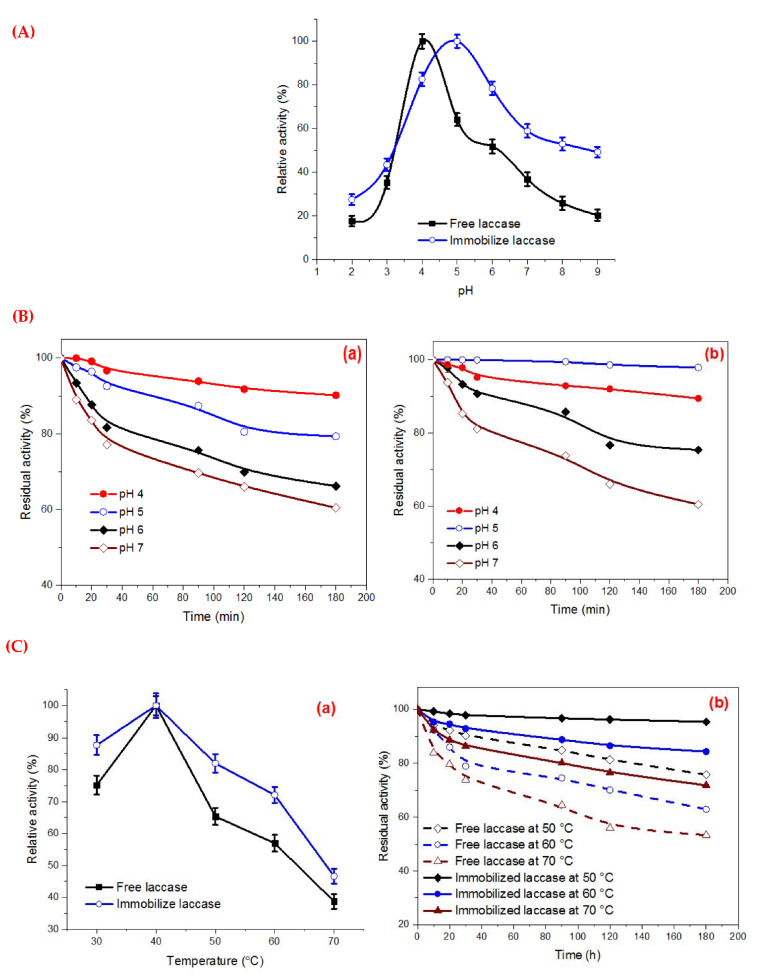
(**A**) Effect of different pH on free laccase and Fe_3_O_4_/3-MPA-S-S-Lac, (**B**) pH stability of (**a**) free Lac and (**b**) Fe_3_O_4_/3-MPA-S-S-Lac (**C**) Effect of different temperatures on free laccase and Fe_3_O_4_/3-MPA-S-S-Lac (**a**), Thermostability profile at 50 °C, 60 °C, and 70 °C for 180 min by free Lac and Fe_3_O_4_-3MPA-S-S-Lac (**b**).

The pH optima of Lac immobilized on the surface of thiol-functionalized Fe_3_O_4_-NPs and the free enzyme were examined by incubating the preparations in the pH range of 2–9 using various buffer systems at constant temperature. The Fe_3_O_4_/3-MPA-S-S-Lac and free laccase showed maximal activity at pH 5.0 and pH 4.0, respectively, according to Figure 7A. The pH stability of the free and immobilized biocatalyst was assayed by incubating the preparations over the range from 4.0 to 7.0 (Figure 7B). In general, the immobilization process led to a remarkable stabilizing effect toward various pH when compared to the free one as a result of the rigidity of the conformational structure upon immobilization, related to microenvironment change [6,31].

#### 3.6.2. Temperature Optima and Thermostability

The profile of the free laccase and Fe_3_O_4_/3-MPA-S-S-Lac activity was performed by incubating the enzyme solution at various working temperatures, i.e., 30–70 °C. Laccase immobilization led to slight brooding of the activity/temperature curve, especially in the temperature range of 50 to 70 °C (Figure 7C(a)). Thermostability is considered one of the critical challenges affecting the application of enzymes as biocatalysts in different industries. Generally, the enzyme immobilization to specific support makes it resistant to drastic conformational variations [6,12,14]. A novel support, particularly nanocomposite, has been widely applied in the enzyme immobilization processes. Thermostability of the free and immobilized laccase was performed by preincubating the biocatalyst preparation without substrate at different temperatures, i.e., 50–70 °C, at constant pH for 180 min. The solution was allowed to equilibrate for 60 min at ambient temperature and the activity was then assayed as described above. The residual activities were detected using the standard assay method. 

In the present study, the immobilized laccase was more stable toward heat denaturation, with only a 5% of laccase activity loss at 50 °C when compared to the free counterpart (Figure 7C(b)). The enhanced thermostability of immobilized enzyme could be assigned to the decline of heat transfer to enzyme microenvironment and the protection of active conformational site as a result of the covalent linking arising between laccase and the thiol functionalized magnetic nanoparticles [6,41]. 

#### 3.6.3. Kinetic Parameters 

Classical Lineweaver-Bürk plots of the free laccase and Fe_3_O_4_/3-MPA-S-S-Lac were used to evaluate the enzymatic kinetic parameters, i.e., V_max_, K_m_, and K_cat_ by using different concentrations of ABTS (non-phenolic substrate) and catechol (phenolic substrate) as synthetic substrate under standard assay. As illustrated in Table 3, the K_m_ values of immobilized laccase for ABTS and catechol were 2.60 and 0.93 mM, respectively, which were lower than the free enzyme (4.15 and 1.3 mM). The V_max_ values of free and immobilized enzymes were augmented to be 29.06, 14.22 and 27.03, 14.90 Umg^−1^ protein for ABTS and catechol, respectively. As noticed in the Table 3, the catalytic affinity (K_cat_/K_m_) of Fe_3_O_4_/3-MPA-S-S-Lac was increased, relative to the free enzyme. The probable reasons for kinetic parameters fluctuations are the protein rigidity, reduction in enzyme flexibility for substrate, diffusional restrictions, and slight structural changes in the substrate-binding pocket after covalent immobilization on Fe_3_O_4_/3-MPA-SH, which as a consequence enhanced the limitations of access among the enzyme and the substrate without affecting the transition state binding, hinting an increased rate of reaction [32,42,43]. 

The diffusional coefficient (D_C_) was calculated by dividing the V_max_ value of the immobilized enzyme over the V_max_ value of the free enzyme and used to express the magnitude of mass transfer using different substrates, namely ABTS and catechol. The diffusional coefficient was respectively found to be 0.92 and 1.05 for ABTS and catechol, which was linked to the easy accessibility (D_C_ value more than 1.0) of the substrate into the immobilized enzyme beads [44,45]. 

#### 3.6.4. Influence of Different Organic Solvents on Laccase Stability

For testing the stability of the free laccase and Fe_3_O_4_/3-MPA-S-S-Lac in different water-miscible solvents, the enzymatic preparations were preincubated in 1 mL of the investigated organic solutions at various concentrations (10–50%, *v*/*v*) for 24 h at ambient temperature (Table 4). The enzyme activity was subsequently determined under standard assay conditions, relative to the enzyme preparations without any organic solvent (controls, 100%).

In the present work, both free and immobilized enzymes exhibited a reduction in laccase activity as the concentrations of organic solvents rose. However, the Fe_3_O_4_/3-MPA-S-S-Lac showed considerably higher activity when compared with the free one at various organic solvent concentrations. Both free and immobilized laccase displayed the highest stability when using different concentrations of acetone as the organic solvent. Similar results have been reported by other investigators [46,47,48].

#### 3.6.5. Operational Stability of Immobilized Laccase 

**Figure 8:**
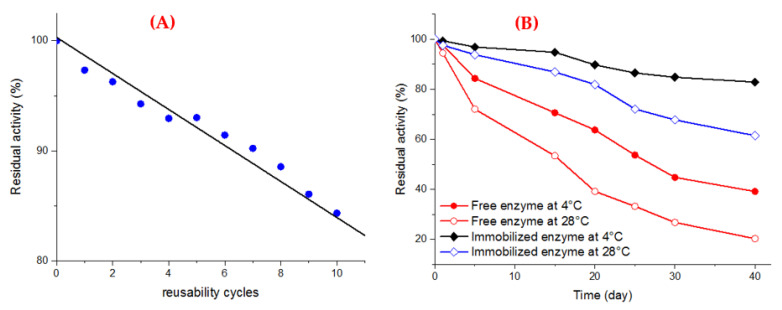
(**A**) Operational stability of immobilized laccase on the thiolated magnetic nanocomposite, (**B**) Storage stability of free laccase and laccase immobilized on the thiolated magnetic nanocomposite at 4 °C (solid) and room temperature (hollow).

The reusability of Fe_3_O_4_/3-MPA-S-S-laccase was examined because of its significant role in reducing the wastewater management processing cost. The reusability of enzyme-magnetic nanocomposite in the oxidation of 0.5 mM ABTS was conducted for 10 consecutive cycles, as illustrated in Figure 8A. By the end of each cycle, the nanocomposite was easily pooled, washed, and reused for the subsequent run. The Fe_3_O_4_/3-MPA-S-S-laccase exhibited remarkable stability while retaining at 84.34% of its initial activity after 10 cycles. The activity loss in repetitive cycles of substrate oxidation might be connected to the repetitive joining of the substrate to active sites of the biocatalyst and hence, influence the binding potency between the enzyme and carrier which is linked to denaturation and inactivation of the enzyme [6,41]. 

#### 3.6.6. Storage Stability 

Generally, the enzyme preparations were not stable when evaluated over different storage periods and storage temperatures, hinting at a consequence effect on the catalytic site and a remarkable loss of its activity. The results in Figure 8B obviously illustrate that the enzyme stability was improved upon immobilization when compared with free laccase through 40 days of storage. The free and immobilized biocatalyst maintained at 4 °C retained 39.2% and 82.9% of their initial activity after 40 days of storage, respectively. Under similar storage conditions, the residual activity of both free and immobilized enzymes was respectively reduced with an approximate loss of 79.7% and 38.5% of its original activity at 28 °C. [44,48], which demonstrated that the immobilized laccases exhibited storage stability higher than the free systems when stored for the same storage time. The superior stability of the immobilized enzyme may be ascribed to the multi-point binding sites of the supports surface to the biocatalysts [32,49].

### 3.7. Biotechnological Application of Free Laccase, Fe_3_O_4_/3MPA-SH, and Fe_3_O_4_/3-MPA-S-S-Laccase for Catalytic Decolorization of Dyes 

**Figure 9:**
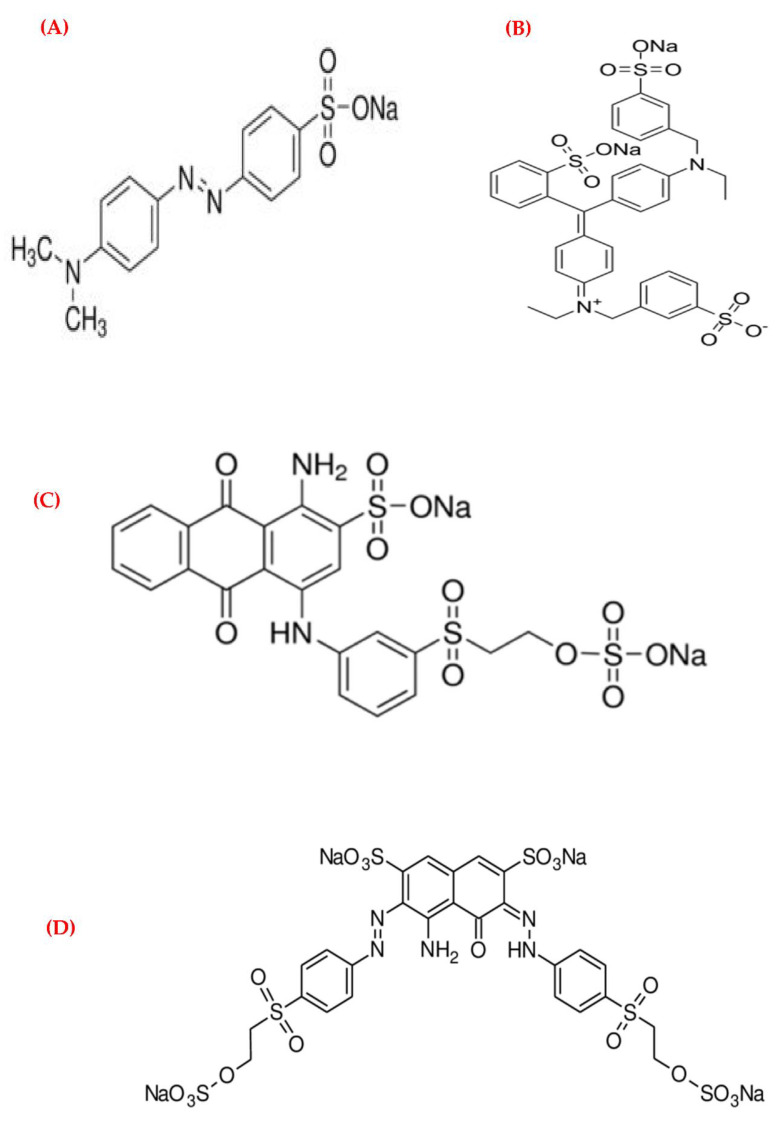
Chemical structures of the target pollutants (dyes): (**A**) Methyl Orange (azo type), (**B**) Brilliant blue (triarylmethane type), (**C**) Remazol Brilliant Blue R (anthraquinone type), (**D**) Reactive Black-5 (diazo type), and redox mediator (1-hydroxybenzotriazole).

The dye decolorizing capability of the free enzyme, Fe_3_O_4_-NP_s_/3MPA-SH, and Fe_3_O_4_-NP_s_/3MPA-S-S-Lac was tested for various textile dyes from the groups of azo (MO), triarylmethane (Bb), anthraquinone (RBBR), and diazo (RB-5). The chemical structure of the target pollutants and the redox mediator are presented in Figure 9. 

The degradation capability of the free preparation without the redox mediator was not detected within 24 h. Meanwhile, the Fe_3_O_4_-NP_s_/3-MPA-SH exhibited 12.51%, 15.67%, 7.14%, and 5.09% removal efficiency of MO, CV, RBBR, and RB-5 in the absence of the redox mediator, respectively. However, the Fe_3_O_4_/3-MPA-S-S-laccase without the redox mediator displayed no appreciable fluctuation in the removal efficiency of the target environmental pollutants, hinting at the importance of the redox mediators in the degradation systems [6] and the role of the adsorption process in the removal of dyes by enzyme-magnetized nanocomposite [1].

**Figure 10:**
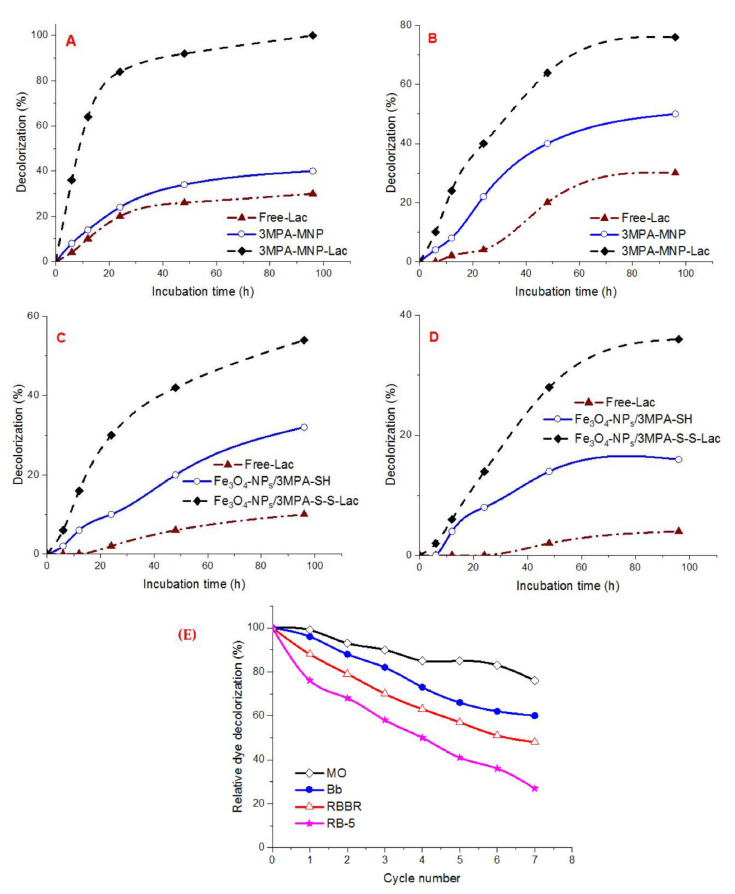
(**A**) Time course of different synthetic dyes (**A**) Methyl Orange, (**B**) Brilliant blue, (**C**) RBBR, (**D**) RB-5 decolorization by free-Lac (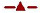), Fe_3_O_4_-NP_s_/3MPA-SH (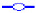) and Fe_3_O_4_-NP_s_/3MPA-S-S-Lac (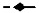), (**E**) Repeated decolorization cycles of environmental pollutants (MO, Bb, RBBR, and RB-5) in the presence of a redox mediator by Fe_3_O_4_/3-MPA-S-S-Lac.

In the presence of the redox mediator (1-hydroxybenzotriazole), the results illustrated a remarkable decolorization capability using Fe_3_O_4_/3-MPA-S-S-laccase (Figure 10A(D)). The decolorization level of MO by free and immobilized enzymes alone was higher than that for the other three investigated dyes in the existence of a 1 mM redox mediator. The decolorization percentage of anthraquinone and diazo by the immobilized enzyme was 30% and 14% within 24 h, respectively. Whereas the triarylmethane decolorization represented 40% within 24 h of incubation (Figure 10A(D)). Furthermore, there was no noticeable decolorization of the anthraquinone dye RBBR and diazo dye RB-5 dye by free preparation and Fe_3_O_4_-NP_s_/3-MPA-SH, compared with Fe_3_O_4_/3-MPA-S-S-laccase. 

The developed system (Fe_3_O_4_/3MPA-S-S-laccase) showed a remarkable decolorization potential, compared to laccase immobilized on different nanosupport systems [1,5,9]. The higher removal efficiency in the case of Fe_3_O_4_/3MPA-S-S-laccase for dyes belonging to different chromophore groups is attributed to either degradation using enzymes or/and biosorption of the synthetic dyes onto magnetized beads [1,6,33]. In comparison with Fe_3_O_4_/3MPA-SH and the free enzyme, the higher decolorization affinity obtained by laccase immobilized on Fe_3_O_4_-NP_s_/3-MPA-SH for different dyes may be linked to the activity of laccase and the availability of adsorption sites on beads with emphasis on amino, thiol, carboxylic, and hydroxyl groups, which improved the adsorption efficiency. Likewise, the steric hindrances reduced the accessibility of sulfonate, hydroxyl, and amino groups on the dye sites to laccases [44,48]. 

#### Reusability Assessment of the Covalent-Immobilized Laccase for Catalytic Decolorization of Dyes 

The immobilized enzymes reusability is a promising feature for wide industrial applications as it reduces the process cost [6]. In the present work, the Fe_3_O_4_/3-MPA-S-S-laccase was employed in seven successive cycles each of 24 h. After the fifth cycle, the relative decolorization rate was above 50%, except for RB-5 (41.02%) (Figure 10E). The removal efficiency of target pollutants was gradually reduced in subsequent degradation cycles. Such a reduction in removal efficiency during additional reuse may be connected to the deactivation of the biocatalyst upon repeated batches, the outflow of biocatalyst from beads at the end of the cycle during washing, and blocking of bead pores by substrate or product [6,44,48]. 

### 3.8. Cytotoxic Effect

**Figure 11:**
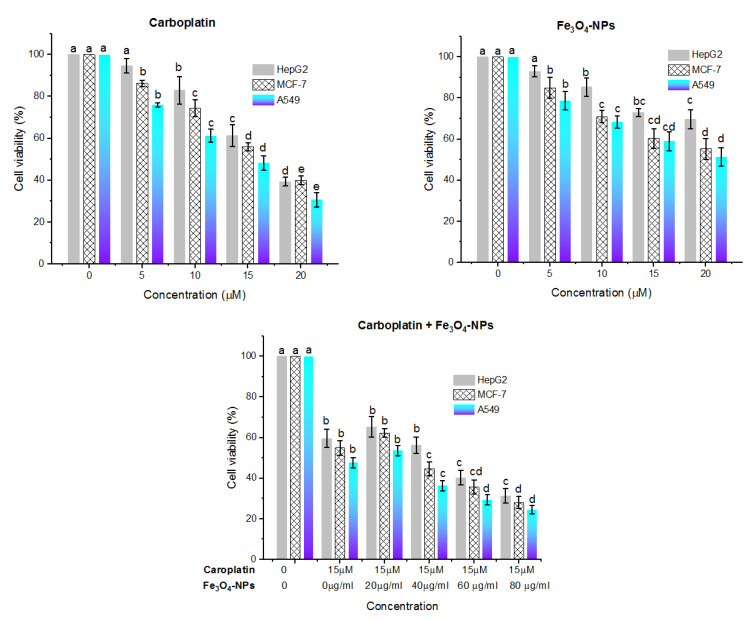
Cytotoxic effect of carboplatin, Fe_3_O_4_-NPs, and combination of carboplatin and Fe_3_O_4_-NPs on the Hep G2, MCF-7, and A549 cell lines. Cell viabilities are detected using MTT assay. Cells are incubated with various concentrations of the tested compound for 24 h at 37 °C. Results are illustrated as means ± standard deviations. Different letters represent significant differences (*p* < 0.05) within various concentrations of the same substance. One-way ANOVA, then Tukey’s HSD test was performed. *n* = 3 independent experiments.

The HepG2, MCF-7, and A549 cells were subjected to different concentrations of carboplatin (5–20 µM) and Fe_3_O_4_-NPs (20–80 µg/mL), alone or in combination (carboplatin and Fe_3_O_4_-NPs), and the cytotoxic effect was determined using MTT analysis. Figure 11 illustrates that the toxicity level of the tested substances (carboplatin and Fe_3_O_4_-NPs) on various cell lines was concentration-dependent (*p* < 0.05). In the MTT assay, the combination of carboplatin and Fe_3_O_4_-NPs showed a reduction in cell viability percent. The IC_50_ values of the carboplatin were 3.6, 3.4, 3.0 µM, however, they were 6.2, 4.1, 3.8 µg/mL for Fe_3_O_4_-NPs. On the other hand, the IC_50_ values of the combination of carboplatin and Fe_3_O_4_-NPs were 3.3, 2.8, 2.4 µg/mL against HepG2, MCF-7, and A549, respectively. Hence, the developed nanoparticles are considered as a promising drug delivery system. These findings are in harmony with [50] who recorded that Fe_3_O_4_-NPs alone diminished the viability of PC-3 and LNCaP cells. It seems likely that the Fe_3_O_4_-NPs cytotoxicity depends on the type of cells tested which may be linked with the various redox state properties. It has been reported that Fe_3_O_4_-NPs can cause cytotoxicity through the generation of ROS which induce damage to DNA, Protein oxidation, and lipid peroxidation [51,52,53]. ROS result from the transfer of electrons to oxygen and their levels are managed by enzymatic and non-enzymatic antioxidants. The high level of ROS in cancer cells plays an important role in metastasis [54]. Excessive increases in ROS to about the threshold, resulted in cell toxicity, making some carcinogenic cells susceptible to induced apoptosis by ROS.

## 4. Conclusions

In the present study, the Fe_3_O_4_/3-MPA-SH hybrid nanocomposite was employed as a novel nanosupport for laccase immobilization. The biogenic synthesized thiolated nanocomposite was characterized by FT-IR, XRD, SEM, and TEM analyses. Interestingly, the immobilization of laccase extracted from *P. expansium* on the thiolated nanosupport displayed superior stability over the soluble biocatalyst at various operating parameters (thermal, pH stability, and storage). The above-developed biocatalyst was applied to the decolorization of synthetic textile dyes in presence of a redox mediator system. The immobilized biocatalyst exhibited a significant removal rate for MO, Bb, RBBR, and RB-5. However, additional research is required to explore the efficiency of Fe_3_O_4_/3-MPA-S-S-Lac for other target pollutants under particular operating conditions. The improved recycling and regeneration of biocatalyst immobilized on the thiolated nanocomposite could be a promising advantage in the decontamination of pollutants. The above results provide insight into the performance of laccase immobilized on a novel nanosupport and suggest its use in wastewater treatment could be described as satisfactory. However, the use of nanomaterial at the industrial scale for environmental pollutant degradation has not been employed at a large scale due to their high cost and poor regeneration. Further research is needed into the use of the developed nanosupport for the immobilization of other enzymes and the application for environmental pollutants’ degradation in real wastewater systems. In addition, the combination of carboplatin and Fe_3_O_4_-NPs showed a remarkable reduction in cell viability (%).

## Figures and Tables

**Table 1:** Purification profile of the laccase obtained from *P. expansium*.

Purification Steps	Total Activity (U)	Total Protein (mg)	Specific Activity (U/mg)	Purification Fold	Yield (%)
Crude enzyme	2254.5	224.64	10.03	1	100
Ammonium sulfate (75%)	1566	49.59	31.94	3.18	69
DEAE-cellulose	951.6	6.422	148.17	14.76	42
Sephadex G-200	625.3	1.885	331.72	33.05	27

**Table 2:** Comparison of thiolated magnetic nanoparticles with other supports for the immobilization of laccase.

Support	Laccase Loading (mg/g)	References
Fe_3_O_4_/3MPA-SH	112	PS
LA-Au/PDA@SiO_2_-MEPCM	50	[37]
Magnetic biochar	27	[32]
Magnetized-chitosan-grafted hallohalloysite nanotube	100	[1]
Magnetic-chitosan	32	[32]
MACS-NIL-Cu-Laccase	47	[38]
Chitosan-functionalized supermagneti cellulose	73	[39]
Sepabeads EC-EP3	32.6	[40]
Dilbeads NK	17.8	[40]

PS: Present study.

**Table 3:** Kinetics of free and immobilized laccase. The parameters were assayed at optimal pH and temperature using different concentrations of catechol and ABTS.

Substrate	Free-Laccase				Fe_3_O_4_-NP_s_/3-MPA-S-S-Laccase			
	K_cat_ (S^−1^)	K_m_ (mM)	V_max_ (U/mg Protein/min)	K_cat_/K_m_ (mM^−1^ S^−1^)	K_cat_ (S^−1^)	K_m_ (mM)	V_max_ (U/mg Protein/min)	K_cat_/K_m_ (mM^−1^ S^−1^)
ABTS	45.05	4.15	29.06	10.87	48.45	2.60	27.03	18.65
Catechol	23.71	1.31	14.22	18.14	24.84	0.93	14.90	26.75

**Table 4:** Stability of the free and immobilized laccase on thiolated magnetic nanoparticles in different organic solvents concentrations.

Residual Activity (%)	Concentrations of Organic Solvents (%)														
	Acetone					Methanol					Ethanol				
	10	20	30	40	50	10	20	30	40	50	10	20	30	40	50
Free laccase	98.6	93.3	89.6	85.7	79.8	87.6	74.3	61.1	46.9	32.2	94.4	88.3	80.5	75.2	63.8
Fe_3_O_4_/3-MPA-S-S-laccase	99.6	96.1	93.6	90.7	84.6	91.1	78.2	65.1	49.8	35.3	96.1	93.9	89.4	84.8	79.3

## Data Availability

The study did not report any data.
